# The determinants of under-5 age children malnutrition and the differences in the distribution of stunting–A study from Armenia

**DOI:** 10.1371/journal.pone.0249776

**Published:** 2021-05-26

**Authors:** Pavitra Paul, Bhanu Arra, Mihran Hakobyan, Marine G. Hovhannisyan, Jussi Kauhanen

**Affiliations:** 1 Institute of Public Health and Clinical Nutrition, University of Eastern Finland, Kuopio, Finland; 2 United Nations Children’s Fund, Yerevan, Armenia; 3 Faculty of Public Health, Yerevan State Medical University, Yerevan, Armenia; University of Botswana, BOTSWANA

## Abstract

Stunting undermines economic growth by perpetuating the vicious cycle of poverty and labour market performance. Studies have captured the trend in stunting and present distributional evidence of policy effects in the country contexts. We identify the determinants of U5 (under 5 years of age) malnutrition for the poor and the Nonpoor and compare the distribution of stunting at four time points (2000, 2005, 2010 and 2015) over a 15-year period between different groups of population. Further, we decompose the gap in malnutrition into causes of differences in stunting between worse-off and better-off socioeconomic groups of the population and estimate the magnitude of distributional differences in stunting between two socioeconomic groups. We also present the inequality trend over time that provides insights into the dynamicity of the effect of different determinants on stunting at different time points. Using 35,490 observations from Armenian Demographic and Health Survey Data [four waves: Year2015,9533; Year2010,8644; Year2005,8919; Year2000,8334], we apply regression-based decomposition method and inequality measures to identify the determinants of malnutrition and distribution of stunting between and within socioeconomic groups. Although the proportional difference in prevalence of stunting between worse-off and better-off children of 13 months and above are reduced by 9.5% in 2015 compared to 2000, the association between socioeconomic position and stunting is statistically significant among children aged 13 months and above in 2000, as well as among children of 36 months and above in 2015. This study demonstrates that the less of socioeconomic distribution of the population, but rather more of the effect from in-country region and settlement of residence are significantly associated with stunting. The approach of our analysis is potentially also a useful tool to generate evidence for decision making towards achieving SDGs 2.2. We conclude that development in childhood is not independent from the distributional effect of region specific development initiatives. Understanding the regional characteristics and resources allocated for the maternal and child health is the necessity to address stunting.

## I. Introduction

Globally, an estimated 151 million (22.2 percent) of children under 5 years of age are affected with stunting [1]. Stunting is the impaired growth and development that children experience from poor nutrition, repeated infection, and inadequate psychosocial stimulation [2]. A stunted child can never reach to full height and his/her brain may not attain its ultimate cognitive capacity [1]. Long term consequences of stunting include reduced somatic and mental development, diminished work productivity and deprived health [3–5]. Grave outcomes in health, social and economic fronts are not only seen in individuals across generations but also on the societies resulting in reduced overall development of the population [4, 5]. Stunting undermines economic growth by perpetuating the vicious cycle of poverty and labour market performance, especially in agrarian societies [6–9].

Various household factors like income, consumption pattern and relationship harmony are related to stunting. Social networks associated with poverty contribute to stunting. Urban and rural settlements are also having differential effects on stunting [10]. Biological factors of stunting are diarrhea, low birth weight, duration of breast-fed, low hemoglobin levels and frequency of episodes of nutritional deficiencies during childhood, and so are the age, height and weight of the mother at the time of conception [11–18]. Social factors include parental education attainment, the region of residence including the settlement (urban or rural location), household size, number of children in the family [12, 15, 16, 19, 20]. Intergenerational influence of stunting along with low birthweight has also been well established [21]. Less number of children in a family is associated with higher and more equal investment of households per child [22].

The most common manifestation of chronic malnutrition is stunting. Poor nutrition in early childhood is one of the main factors for stunting apart from frequent childhood infections, micronutrient deficiencies, the neighbourhood environment, and poor maternal nutritional status [23–25]. Stunting is considered an irreversible outcome of inadequate nutrition and repeated infection during the first 1,000 days of a child’s life [5, 26, 27]. The adverse effects appear to be stronger for children who are exposed to malnutrition during first two years of life [28]. Malnutrition impedes development. Sustainable Development Goals (United Nations General Assembly, date 25 September 2015), target 2.2. aspires to end malnutrition by 2030.

Studies [29, 30] have documented that children from higher socioeconomic strata (SES) have relatively lower risk of stunting compared to their lower SES counterparts. A multi-country study using Demographic and Health Survey (DHS) data found that stunting is three times more likely among children in the worse-offs than among those in the better-offs [31]. Another study that examined socioeconomic inequality in child nutrition among twenty developing countries found that eighteen countries had statistically significant inequalities in both stunted and underweight children.

The equity considerations provide insights into the interplay of the social determinants of stunting [32–34]. The importance of investigating changes in health by SES, and other dimensions such as urbanisation and educational background is well founded [35]. The UNICEF [36] conceptual framework of undernutrition has identified poverty and food insecurity, maternal and child care practices, limited access to health services, poor health environment (water, sanitation, and hygiene), gender inequities, and limited education as the underlying determinants of undernutrition. Hovhannisyan et al. [37] found that socioeconomic position of household, child’s length at birth, duration of breast-fed and food diversity predict child’s malnutrition in Yerevan (Armenia). A study by Balalian et al. [38] found the effect of intake of diverse food on stunting in Tavush (Armenia). Factors like parents’ academic and social skills, health seeking behaviours, and quality of household and neighbourhood, in regard to support and cohesiveness influence child development [39].

Few studies have captured the trend in stunting and present distributional evidence of policy effects in the country contexts. We identify the determinants of U5 (under 5 years of age) malnutrition for the poor and the Nonpoor and compare the distribution of stunting at four time points (2000, 2005, 2010 and 2015) over a 15-year period between different groups of population. The uniqueness of our approach is that we (1) decompose the gap in malnutrition into causes of differences in stunting between worse-off and better-off socioeconomic groups of the population and (2) estimate the magnitude of distributional differences in stunting between two socioeconomic groups. Further, we recognise the asymmetric effects of the determinants and thereby, the use of advanced analysis strategy has captured not only the inequality trend over time but also provides insights into the dynamicity of the effect of different determinants on stunting at different time points.

Republic of Armenia is a former Soviet Union country which was highly industrialized, with a centralized healthcare system. After the breakdown of the Soviet Union in 1991 and with few catastrophic events in recent times, the poor capacity and the reduced capability of the health system have led to a deteriorating nutritional status and health of the population [40, 41].

Armenia is divided into 11 administrative districts called ‘Marzes’ including Yerevan. Armenia is gradually making progress in development through ongoing social, economic, political transformations [40, 41]. However, the global trend of stunting [42] is not observed in Armenia.

This study unfolds the gap in stunting between worse-off (poor) and better-off (Nonpoor) Armenians. Our main finding is that it is not the socioeconomic position of the household, but rather the effect of region (and settlement) of residence is having a strong association with stunting (malnutrition) in Armenia.

This article proceeds as follows: section II describes the data and methods; section III and section IV present the results and the discussion respectively; and section V concludes with articulation of important policy lessons.

## II. Data and methods

We used data from the DHS program of the U.S. Agency for International Development (USAID), conducted in collaboration with the Armenian government, and the Armenian Demography and Health Survey (ADHS). The Armenia Population and Housing Census sampling frames were used for data collection, making the datasets nationally representative [43].

The size of the representative sample was equal from each Marzes (administrative districts). The sample was a two-stage stratified cluster sample–first stage defined rural and urban from the list of enumeration areas covering the whole country and the second stage selected the households for participation in the survey. Sampling weights were based on sampling probabilities calculated separately for each sampling stage and for each cluster.

The files [https://dhsprogram.com/What-We-Do/survey-search.cfm?pgtype=main&SrvyTp=country] of different datasets (household, mother and children) were merged for each wave (2015, 2010, 2005 and 2000). The total number of observations used in the study was 35,490 from the four waves [Year2015:9533; Year2010:8644; Year2005:8919; Year2000:8334]. The respondents came from 5833 households. Table 1 presents the descriptive statistics of the children in the study.

**Table 1:** Mean height and mean weight by gender and age-group–U5 children, study country–Armenia.

Year ->		2000	2005	2010	2015
Gender	Mean height (centimeter)				
Boys (N = 3445)	Age groups				
	0-12months	65.83(8.02)	65.83(8.16)	65.14(8.73)	66.62(8.58)
	13-36months	84.12(6.78)	86.04(8.29)	83.51(7.89)	86.73(8.55)
	37-59months	98.20(6.19)	101.02(9.06)	98.01(8.77)	102.39(7.85)
	Mean weight (kilogram)				
	0-12months	7.80(2.40)	7.64(2.15)	7.59(2.31)	8.02(2.37)
	13-36months	12.54(1.96)	12.55(2.15)	12.58(2.02)	13.09(2.3)
	37-59months	16.38(2.16)	16.53(2.95)	16.17(2.71)	17.12(3.35)
	***N***	931	775	766	973
Girls (N = 2908)	Mean height (centimeter)				
	0-12months	65.70(7.20)	63.73(9.46)	65.26(8.78)	66.33(8.49)
	13-36months	83.71(6.65)	84.24(9.34)	82.33(8.58)	85.68(7.99)
	37-59months	98.35(6.56)	100.20(8.31)	97.82(8.61)	101.71(7.54)
	Mean weight (kilogram)				
	0-12months	7.49(2.08)	7.2(4.32)	7.34(2.28)	7.76(2.26)
	13-36months	12.20(1.93)	11.77(2.26)	11.78(2.12)	12.44(2.14)
	37-59months	15.87(2.12)	15.93(2.78)	15.83(2.77)	16.67(3.28)
	***N***	793	698	664	753

Figures in parenthesis indicate standard deviation.

Although inconsistent variations were registered over the years, the mean height and the mean weight were marginally higher for both genders in 2015 compared to 2000. The boys were weighing relatively more than the girls in all age-groups and in every year (Table 1).

### Study variables

The outcome variable of interest used was stunting in children under five years of age. Stunting is the measure of Height for Age Z-score (HAZ) with values below two standard deviations of WHO [44] median value—HAZ score indicates long-term effects of malnutrition [45].

Explanatory variables were selected based on prior knowledge from the literatures and availability in the data. Individual factors included were (1) child level factors such as age, gender, birthweight, duration (in months) of breast fed, suffering from diarrhea (during the last two weeks prior to the survey), and (2) maternal factors (mother’s age at first birth, mother’s education level–three levels, Primary, middle school and high school completed, and Rohrer’s index). Rohrer’s index is an anthropometric measurement defined as weight in kilograms divided by height in meters cubed [weight / (height)^3^] and serves similar purpose to BMI, here it considers body as a three-dimensional entity as opposed to BMI, where body is measured as a two-dimensional entity [46].

Household factors included were the number of members in the household, total number of children in a family, and reported incidence of violence in the family. In addition, the neighbourhood factors included were the type of settlement (urban or rural), and region of residence in the country (geographic location– 11 regions: Aragatsotn, Ararat, Armavir, Gegharkunik, Lori, Kotayk, Shirak, Syunik, Vayots dzor, Tavush, and Yerevan). Birthweight, Rohrer’s index, or mention of diarrhea were missing in the 2010 data. The indicator of affluence used in the study was the ‘Wealth index’.

Wealth index is a composite measure of a household’s cumulative living standard. It is calculated using easy-to-collect data on a household’s ownership of selected assets, such as televisions and bicycles; materials used for housing construction; and types of water access and sanitation facilities [47]. The population is distributed into five quintiles based on the wealth index of the households [47].

We distribute the households as ‘poor’ and ‘Nonpoor’ based on the ‘Wealth index’ [first 2 quintiles i.e. poorest and 2^nd^ poorest are grouped as “poor” and the remaining three quintiles i.e. middle, 2^nd^ richest and richest, “Nonpoor”].

### Data analysis

#### Step 1

We examined the systematic differences of regression coefficient vector ‘β’ (Eq 1) between children from poor and Nonpoor households. Our poverty grouping variable is poor, which takes value of 1 if the child is from poor household.

$$y_{i}=\{\begin{array}{c} \beta^{poor}x_{i}+\varepsilon_{i},\;if\;poor\\ \beta^{Nonpoor}x_{i}+\varepsilon_{i},\;if\;Nonpoor \end{array}$$ Eq 1

*y*, our outcome variable i.e. HAZ was explained by a vector of determinants, *x* from the regression model where the vectors of ‘β’ parameters include intercepts. In addition, the Nonpoor are assumed to have a higher mean of *x*. In the case of the poor, we read off the equation for the poor above *x*^*poor*^, giving a value of *y* equal to *y*^*poor*^. In the case of the Nonpoor, we read off the equation for the Nonpoor above *x*^*Nonpoor*^, giving a value of *y* equal to *y*^*Nonpoor*^.

The gap between the mean outcomes, *y*^*Nonpoor*^ and *y*^*poor*^ is equal to

$$y^{Nonpoor}-y^{poor}=\beta^{Nonpoor}x^{Nonpoor}-\beta^{poor}x^{poor},$$ Eq 2

where *x*^*Nonpoor*^ and *x*^*poor*^ are vectors of explanatory variables evaluated at the means for the Nonpoor and poor respectively. The conditional expectations of the error terms in Eq 1. are zero, assuming exogeneity. Thus, the gap in *y* between the poor and the Nonpoor can be thought of as being due in part to (i) differences in the intercepts, (ii) differences in x_1_ and β_1_, differences in x_2_ and β_2_, ………, differences in x_n_ and β_n_.

Estimates of the difference in the gap in mean outcomes were obtained by substituting sample means of the *x*′*s* and estimates of the parameters *β*’s into Eq 2.

Our mean VIF (variance inflation factors) was 1.3 and so, there was no multicollinearity (correlation between the predictors).

#### Step 2

Oaxaca’s decomposition

Here, we examine how much of the overall gap or the gap specific to any one of the *x*′*s*) is attributable to (i) differences in the *x*’s (often, called the explained component) rather than (ii) differences in the β’s often, called the unexplained component.

$$y^{Nonpoor}-y^{poor}=\Delta x\beta^{Nonpoor}+\Delta \beta x^{poor},$$ Eq 3

*where*

$$\Delta x=x^{Nonpoor}-x^{poor}\;and\;\Delta \beta =\beta^{Nonpoor}-\beta^{poor}.$$

The differences in *x*′*s* are weighted by the coefficients of the poor group and the differences in the coefficients are weighted by the *x*′*s* of the Nonpoor group; thus, we partitioned the gap in outcomes between poor and Nonpoor into a part attributable to the fact that the poor have worse *x*’s than the Nonpoor.

Eq 3. is a case from general decomposition [48]

$$y^{non-poor}-y^{poor}=\Delta x\beta^{poor}+\Delta \beta x^{poor}+\Delta x\Delta \beta =E+C+CE,$$

i.e. gap in outcome was from a gap in endowments (E), a gap in coefficients (C), and a gap arising from the interaction of endowments and coefficients (CE).

We have tested regression-based models for goodness-of-fit and heteroskedasticity. We found variance of residuals nonconstant and so, we used an exponential function of covariates; likelihood–ratio test confirms the fitness of the data in the model of the variance.

Finally, we compared and quantified the socioeconomic (poor vs. Nonpoor) gradient of stunting in relative (relative slope index of inequality in stunting) and absolute (slope index of inequality in stunting) terms and examined the trend over time [49]. Children who fall below negative two standard deviations (−2 SD) are classified as stunted.

Concerning the relative and the slope indices of inequality, an important consideration is that with these indices, it is not the socioeconomic group itself that is important, but its relative size and position in the population, measured through the socioeconomic rank *x*. To define Relative index of inequality (RII) in stunting, we considered log-linear models of the form *f*_*β*_(*x*) = *y*_0_exp(*β*_*x*_), indexed by parameter *β*, with *y*_0_ > 0 being a nuisance parameter [50, 51]. Setting *y* = *f*_*β*_(*x*), the socioeconomic gradient may be characterised by the factor exp(*β*), which indicates the magnitude of the linear association between *x* and *y* in relative terms and its direction–above 1 if the association between *x* and *y* is positive and below 1 if, negative. We defined *RII* = exp(β*), where β* is the parameter that yields the best approximation of the association between *x* and *y* by a log-linear model. RII is estimated by fitting a multiplicative Poisson model. The estimate of the RII is given by $\hat{RII}=exp(\hat{\beta })$, where $\hat{\beta }$ is obtained by maximum likelihood.

Slope index of inequality (SII) in stunting was calculated as 2*ASSR*(RII-1)/(RII+1), where ASSR is age standardized stunting rate for gender, region and year combination [49]. The goodness of fit test statistics confirmed the adequacy of the model used.

## III. Results

Stunting was associated with socioeconomic position (SEP) of the households in almost every study year, but such association was significant for children in the age-group of 37–59 months in 2015 and in 2000, and for children in age-group of 13–36 months in 2005 and in 2000 (Table 2). We could not find any consistent pattern of distribution of stunted children by SEP of the households but relatively a greater number of children in age-group of 37–59 months were stunted in the poorest households in 2015 and in 2000. Although the association between SEP and stunting was not significant, substantially more representation of stunted children in age-group of 37–59 months was from the households of middle SEP in 2010 and from that of the richest SEP in 2005.

**Table 2:** Percentage distribution of stunting by socioeconomic position (SEP) of the household, study country–Armenia.

	2000			2005			2010			2015		
	0–12	13–36	37–59	0–12	13–36	37–59	0–12	13–36	37–59	0–12	13–36	37–59
	months	Months	months	Months	Months	months	Months	Months	months	Months	Months	months
	(%)	(%)	(%)	(%)	(%)	(%)	(%)	(%)	(%)	(%)	(%)	(%)
Poorest	1.31	5.44	5.14	1.82	4.07	2.71	2.87	4.4	3.08	1.56	1.4	2.98
2nd poorest	1.97	3.81	4.99	1.09	1.74	2.26	0.64	3.49	3.3	2.6	0.98	1.49
Middle	0.66	2.18	2.42	1.82	4.07	1.36	2.23	3.67	6.59	2.08	2.24	1.34
2nd richest	1.31	0.91	1.36	2.18	2.71	2.26	2.23	3.85	3.96	1.04	1.54	0.89
Richest	0.33	1.81	1.66	0.73	0.78	11.58	2.55	1.83	2.64	1.56	0.84	0.45
χ2 test (p-value)	0.577	0.036	0.012	0.727	0.008	0.598	0.054	0.281	0.188	0.873	0.219	0.002
***N*** (households)	***305***	***551***	***661***	***275***	***516***	***442***	***314***	***545***	***455***	***384***	***713***	***672***

Values indicate percentage of stunted children only for that age group expressed in months for the corresponding year.

Table 3 presents regression coefficient (*βs*) and interaction of level of affluence (poor) with a set of independent variables (*X*s) for HAZ. The relationship between HAZ and child’s age differed significantly between poor and Nonpoor in year 2000. Girls had a lower probability of being malnourished in 2000. The probability of malnutrition in poor households were less with a high Rohrer’s index in 2000. High birth weight significantly reduced the probability of malnutrition in all the years. More duration of breast fed and higher age of the mother at first child birth were having positive contribution for a better HAZ-score in poor households in 2015. Region of residence were having a significant association with probability of malnutrition among children from poor households in 2015 and so, was the effect of violence in the poor households.

**Table 3:** Determinants of U5 malnutrition (measured anthropometrically through ‘height-for-age’[HAZ] scores) for the poor and the Nonpoor, study country–Armenia.

Variables	2000	2005	2010	2015
	(*β*)	(*β*)	(*β*)	(*β*)
Poor [poor = 1; Nonpoor = 0]	6.460	-27.839	13.676	31.296
LogAge	-24.619***	-0.376	-11.615	-3.081
Poor*LogAge	0.054	0.017	0.053	-0.146
Gender [male = 1]	21.563*	-2.093	8.780	16.113
Poor*Gender	-0.0826	-0.006	0.191	0.064
Birthweight	0.058***	0.063***	-	0.055***
Poor*Birthweight	-0.000	-0.000	-	-0.000
Duration of breast fed	0.302	0.126	-	0.324
Poor*Duration of breast fed	-0.001	-0.0103*	-	0.006**
Suffering from diarrhoea	6.378	6.925	-	15.455
Poor*Suffering from diarrhoea	0.048	0.148	-	-0.251
Mother’s age at 1st child birth	0.769	-0.558	0.506	1.005
Poor*Mother’s age at 1st child birth	-0.007	-0.022	0.045*	0.048***
Mother’s highest education [comparison: primary education]	2.992	24.831	13.000	13.371
Poor*Mother’s highest education	0.015	0.322	-0.151	0.021
Rohrer’s index	0.023	-0.015	-	-0.010
Poor* Rohrer’s index	0.001**	-0.000	-	-0.000
Household size	-0.123	3.537	0.966	-2.447
Poor*Householdsize	0.0227	-0.051	-0.089*	-0.025
Number of children in the household	-11.93	-0.017	-5.480	7.339
Poor*number of children in the household	0.0336	-0.032	0.153*	0.077
Violence in the household [1 = yes]	-3.652	-20.595	-2.168	39.903*
Poor*violence in the household	-0.163	-0.058	0.094	0.371*
Region of residence [comparison: Aragatsotn]	0.716	4.777**	2.686	-5.961***
Poor*region of residence	-0.0342	0.040	-0.042	-0.143***
Settlement of residence [urban = 1]	-5.270	-17.155	-4.099	9.522
Poor*settlement of residence	-0.390*	0.379**	-0.340*	0.259
Constant	9.616***	9.946***	10.012***	9.916***
LR test- χ2	0.022	0.009	0.006	0.000
*N*	1367	1135	1232	1711

*:p<0.05

**:p<0.01

***:p<0.001

‘-‘: data not available.

‘*’: interaction.

Table 4 reports difference of mean values of HAZ between U5 Children from poor and Nonpoor households, and shows the contribution attributable to the gaps in endowments (E), the coefficients (C) and the interaction effects (CE). The malnutrition gap between Nonpoor and poor was neither uniform nor followed any definite trend. Although the gap was reduced by almost 80% in 2010 compared to 2000 in our study population, we found a spike by 88.32% in 2015 from 2010. Explained part accounted majority of the gaps in malnutrition between poor and Nonpoor U5 children in 2000 and 2005 but the unexplained gaps, in other years. Endowment effect was maximum in 2000 (42% higher than 2015), interaction effect was maximum in 2000 followed by 2015 and coefficient effect, in 2010. Birthweight in 2000, settlement of residence in 2005 and 2010, and mother’s highest education in 2015 explained maximum gap in malnutrition followed by number of children in household 2000 and in 2010, region of residence in 2005 and in 2015; and settlement of residence in 2000, region of residence in 2010, mother’s highest education in 2005 and birth weight in 2015 in order of extent of contribution that defined the explained gaps in malnutrition between poor and Nonpoor.

**Table 4:** Gaps and decomposition of gaps in U5 malnutrition, study country–Armenia.

	2000	2005	2010	2015
Malnutrition gap (Nonpoor-poor)	33.820	18.798	6.835	12.872
Endowment effects	35.128	22.270	-11.724	24.751
Coefficient effects	15.295	0.949	23.067	7.465
Interaction effects	-16.603	-4.421	-4.507	-19.344
Unexplained part (%)	15.295 (45.2)	0.949 (5.1)	23.067 (337.5)	7.465 (58)
Explained part (%)	18.525 (54.8)	17.849 (94.9)	-16.231 (-237.5)	5.407 (42)
Variables explaining the gaps (individual x’s contribution to overall explained gap				
LogAge	-0.317	0.003	0.569	0.074
Gender	-0.394	-0.051	-0.076	0.138
BirthWeight	5.971	-2.426	-	3.566
Breast fed duration	0.944	-0.044	-	-0.229
Suffering from diarrhoea	0.124	-0.008	-	-0.174
Mother’s age at 1st child birth	0.612	-0.479	-2.545	0.765
Mother’s highest education	0.564	3.862	1.084	4.608
Rohrer’s index	-0.070	0.052	-	-0.327
Household size	0.068	-0.715	2.572	1.380
Number of children in the household	4.496	0.006	-3.708	-0.974
Violence in household	0.641	3.093	0.074	-1.515
Region of residence	2.187	6.295	-2.680	4.313
Settlement of residence	3.699	8.260	-11.519	-6.217

‘-‘: data not available.

**Fig 1:**
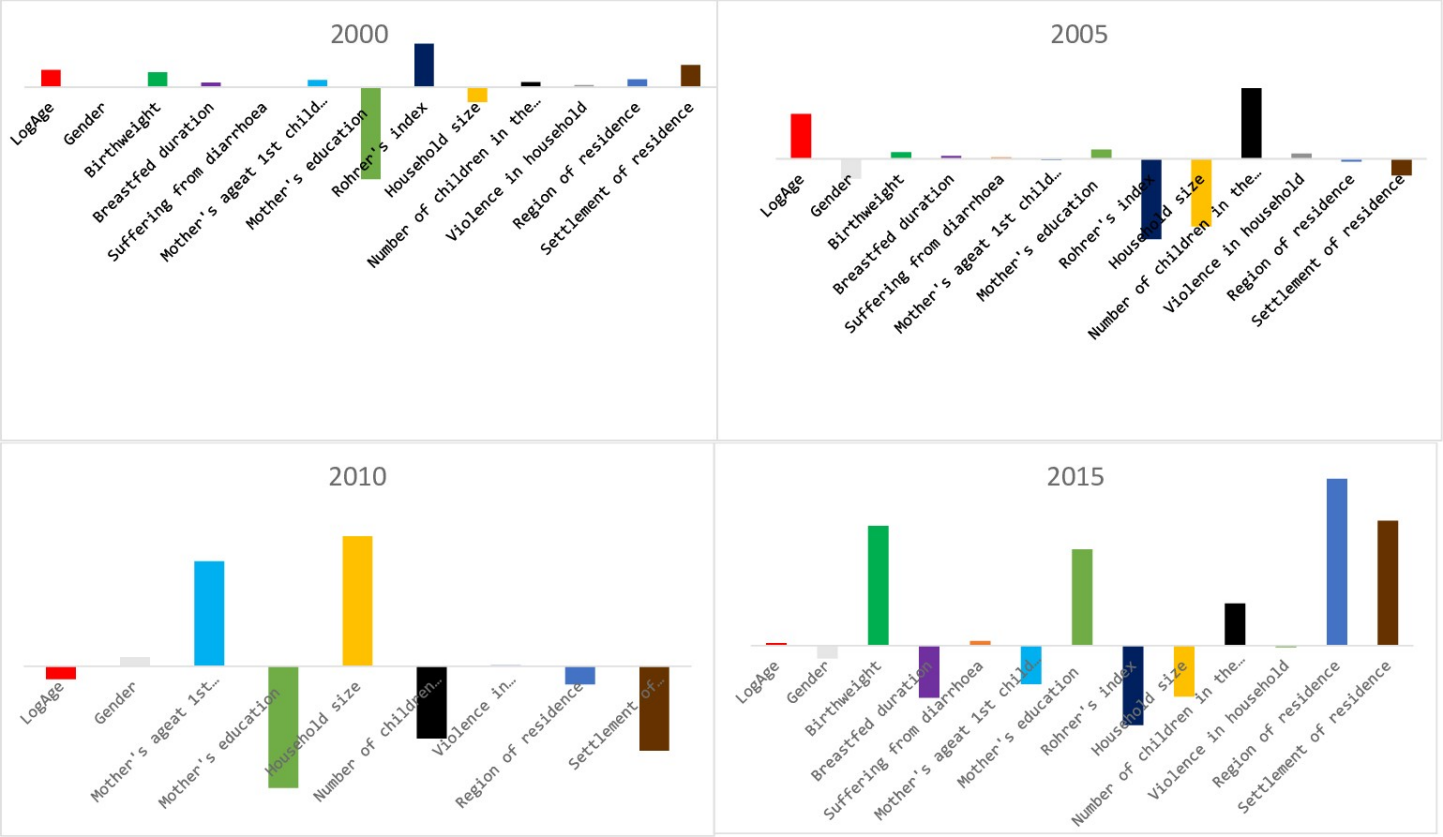
Overall unexplained gap (subtraction of predicted value for the poor from the predicted value for the non-poor) for each explanatory variable, study country–Armenia.

The contributions of explanatory variables for unexplained part of the gap in malnutrition between the children of Nonpoor and poor households were not consistent at each time point over the 15-year study period. Rohrer’s index in 2000; almost in equal proportion, Rohrer’s index, household size and number of children in the household in 2005; mother’s age at 1^st^ child birth, mother’s education level, household size, number of children in the household and settlement of residence in 2010; and birthweight, mother’s education level, Rohrer’s index and settlement of residence in 2015 could be considered to define the unexplained gap in malnutrition between Nonpoor and poor households. In addition, the substantial contribution of region of residence in 2015 could not be ignored (Fig 1) for explaining the unexplained gap.

With 95% confidence the risk of stunting was 2.5 times higher in the poor group compared to the Nonpoor group in 2000, and the difference was statistically significant, while in other years the observed relative risks (between 0.68 and 2.9 in 2005; between 0.4 and 1.32 in 2010; between 0.58 and 2.4 in 2015) were not statistically significant. Statistically significant difference in the absolute risk of stunting was evident in 2000 disfavouring poor group. Although not consistent, the differential effect of region (s) on the magnitude of inequality in stunting was revealed at each time point during the study period except in 2005 (Table 5).

**Table 5:** Socio economic gradient of stunting—relative index of inequality (RII) and slope index of inequality (SII) and effect of region (s) on magnitude of inequality in stunting, study country–Armenia.

	2000		2005		2010		2015	
RII-Poor(95% CI)	2.371*(1.16–4.86)		1.409(0.68–2.93)		0.745(0.42–1.32)		1.184(0.58–2.42)	
SII-Poor(95% CI)	0.110**(0.03–0.19)		0.038(-0.04–0.11)		0.050(-0.16–0.36)		0.013(-0.04–0.07)	
Regions	RII-Poor	SII-Poor	RII-Poor	SII-Poor	RII-Poor	SII-Poor	RII-Poor	SII-Poor
Ararat	1.789	0.070	0.984	-0.003	1.158	0.042	3.541**	0.102**
Armavir	1.023	0.044	0.485	-0.069	0.675	-0.80	2.584*	0.062*
Gegharkunik	3.608***	0.232***	1.293	-0.038	0.895	-0.026	1.388	0.140
Lori	1.538	0.049	0.650	-0.047	0.704	-0.073	1.122	0.003
Kotayk	1.197	0.027	0.500	-0.66	0.415**	-0.146***	1.017	-0.000
Shirak	2.994**	0.158**	0.877	-0.017	0.583	-0.103*	1.212	0.007
Syunik	2.301*	0.102*	0.497	-0.066	1.226	0.059	3.303**	0.092***
Vayots dzor	1.356	0.033	0.590	-0.55	0.120***	-0.220***	2.658*	0.065*
Tavush	1.241	0.023	0.697	-0.41	0.565	-0.108*	1.674	0.026
Yerevan	1.187	0.029	1.187	0.021	0.364***	-0.163***	1.587	0.022

*:p<0.05

**:p<0.01

***:p<0.001

## IV. Discussion

With our objectives to unfold the gap in stunting between worse-off (poor) and better-off (Nonpoor) Armenians and to identify the differential effects of the determinants over time on two different socioeconomic groups, we found that in year 2000, stunting was more prevalent among children aged 12 months and above in worse-off households, while the relative proportion of the stunted children aged 36 months and above was higher in better-off households in 2005 and 2010. Further, the observed reduction of stunting among children of 12 months and above by almost 6.3% in worse-off households and by almost 1.5%, in better–off households over a period of 15 years followed the global trend of stunting [42]. But such trend was not consistent in Armenia during the study period, there was a spike in 2010 (a hike by almost 5% compared to 2005)—percentage of stunting (U5): 13.05 (2000); 10.95 (2005); 16.44 (2010) and 7.41 (2015). Such aberration could be a consequence from the global economic downturn effect on Armenia as well [52]. Although the proportional difference in prevalence of stunting between worse-off and better-off children of 13 months and above were reduced by 9.5% in 2015 compared to 2000, the association between SEP and stunting was statistically significant among children aged 13 months and above in 2000, as well as among children of 36 months and above in 2015. Such finding could be explained by the fact that stunting becomes evident after 1000 days of child’s life [26].

Although high birthweight, and duration of breastfed favoured children from worse-off households in 2005, these variables were not available in our data for 2010. This makes it difficult to make conclusions from just one time point. Our data could not establish a definite trend of the interaction effect between affluence (poor vs. Nonpoor) and each of the variables chosen on stunting, however, the effect of birth weight (birthweight variable was not available in 2010) and that of the settlement of residence (except in 2015) were consistently observed at each time point during the study period. The significant effect of birthweight followed the findings from earlier studies [17]. The effect of settlement of residence in our study supported the findings of Reyes et al. [10] and Janevic et al. [16]. Rural settlement of residence favoured the better-off in 2005 but a reversed association was seen in 2010 (Table 3). Contrary to the earlier study [20], we could not find any effect of mother’s education [15] on stunting in Armenia, but mother’s age at first child birth showed a significant and direct association with stunting in worse-off households in 2010 and 2015.

We decomposed the gap in malnutrition to find the causes of differences in stunting between worse-off and better-off socioeconomic groups in Armenia. The model explained a larger part of the malnutrition gap in 2000 and 2005 but could not for 2010 and 2015. The explained part accounted about 95% of the malnutrition gap between worse-off and better-off in 2005, while the major part of such gap remained unexplained in 2010. The causes of differences in stunting between worse-off and better-off socioeconomic groups were not consistent during the study period–birthweight was most important in 2000, but mother’s educational level in 2015. In general, the settlement type and the region of residence contributed substantially to the malnutrition gap in almost all years of our study (Table 3). Endowment effect in malnutrition gap was found to be tapered down over the time. Furthermore, Rohrer’s index, region and settlement of residence were primarily attributable to the unexplained gap in malnutrition between better-off and worse-off households. Although earlier studies [37, 38] identified different determinants, including patterns of food intake, on stunting in Armenia, our findings point out to the distributional effects of region and settlement of residence on stunting in this country, during recent times.

Finally, when estimating the magnitude of inequality (Table 5) in stunting between two socioeconomic groups and examining the inequality trend over time, we found that the inequality in stunting was significant as was the difference in stunting between the children in worse-off and better-off groups in 2000. Although inequality was evident in 2000, such vulnerability disfavouring the worse-off group was not significant in other years. Effect of region of residence on HAZ–score was found significant in 2005 and 2015 albeit in different direction (Table 3), however, the poor households living in Gegharkunik, Shirak and Syunik in 2000; Kotayk, Vayots dzor and Yerevan in 2010; and Ararat, Armavir, Syunik and Vayots dzor in 2015 were having significant higher risk of having stunted children. A significant difference in the absolute risk of stunting was found in children from Kotayk, Shirak, Vayots dzor, Tavush and Yerevan in 2010 disfavouring Nonpoor. Such observation for 2010 can be explained by the fact that almost 172,000 additional Armenians were pushed below poverty line in 2010 (Armenian economy shrunk by 5.8% in 2009; World Bank).

Never withstanding the inconsistent effect of Rohrer’s index and reported incidence of violence in the family, our study established the effect of region (and settlement) of residence on stunting in Armenia. Moreover, the framework used in our study is replicable across contexts, albeit our study is having also some limitations. The paucity or lack of similar variables in the data (different for 2010) could not allow us to examine the effect of Rohrer’s index on stunting. The representation of respondents was about 8% for each geographical region, except for Aragatsotn (about 10%) and the capital region, Yerevan (about 17%). The representation of households also remained the same in each of the subsequent years. We could not, however, examine the effect of social mobility of the household and what kind of impact on wealth index of the household with the arrival of a new child may have. Thus, we were not able to delineate the effects of such phenomena in this study of a 15-year period, when also the overall development of Armenia was in a transitional path [40, 41]. We did not have information on how similar the scope of antenatal care over time in different regions (settlement of residence), maternity service accessibility and utilisation, and perceived quality of services available and consumed for different socioeconomic groups. Further, limited observations did not allow us to examine the role of parent’s occupation, or the possible effects of infectious diseases and anemia on stunting.

## V. Conclusion

This study examines stunting from the lens of socioeconomic position of the household, distributional effect of different determinants, and the region (and settlement) of residence in the country. Wealth index derived from household’s cumulative living standard, represented by ownership of durable assets, materials of housing construction, and types of water access and provision of sanitation facilities reflects the socioeconomic position of the household. It is not the socioeconomic position of the household, but rather the effect of region (and settlement) of residence that is having a substantial association with stunting (malnutrition) in Armenia. In the light of the decentralised structure of Armenian health system, dominance of out-of-pocket payment (including “gratuities” to doctors) for primary level healthcare services, and prevalence of substantial differences in healthcare infrastructure between regions and geographies [36, 53], our findings require further validation from regional differences in accessibility and affordability to healthcare services, differential consumptions in antenatal care services and in-country distributional differences of maternal and child health initiatives. This study demonstrates that development in childhood is not independent from the distributional effect of region specific development initiatives.

One potential extension of our study is to incorporate regional characteristics and resources allocated for the maternal and child health and reorient the analysis towards geographical regions for designing effective policies for addressing stunting. The approach of our analysis is a useful tool to generate evidence for decision making towards achieving SDGs 2.2.
